# Child Amputee Prosthetics Project—Prosthesis Satisfaction Inventory (CAPP-PSI): Validation of Italian Version in Children with Upper Limb Amputation

**DOI:** 10.3390/children12020130

**Published:** 2025-01-24

**Authors:** Luigino Santecchia, Gessica Della Bella, Francesca Caspi, Paola Luttazi, Lorenzo Pochiero, Fabrizio Taffoni, Giordana Mariani, Marco Gaudenzi, Donatella Valente, Marco Tofani

**Affiliations:** 1 Orthopedic Unit, Hand Surgery and Orthoplastic Service, Bambino Gesù Children’s Hospital, IRCCS, 00165 Rome, Italy; luigino.santecchia@opbg.net; 2 Management and Diagnostic Innovations & Clinical Pathways Research Area, Neurorehabilitation and Adapted Physical Activity Day Hospital, Bambino Gesù Children’s Hospital, IRCCS, 00165 Rome, Italy; gessica.dellabella@opbg.net (G.D.B.); paola.luttazi@opbg.net (P.L.); lorenzo.pochiero@opbg.net (L.P.); giordana.mariani@opbg.net (G.M.); gaudenzi.ma@gmail.com (M.G.); 3 School of Occupational Therapy, Sapienza University of Rome, 00158 Rome, Italy; caspifrancesca@gmail.com; 4 Laboratory of Advanced Robotic and Human-Centered Technologies, Campus Bio-Medico University of Rome, 00128 Rome, Italy; f.taffoni@unicampus.it; 5 Department of Human Neurosciences, Sapienza University of Rome, 00158 Rome, Italy; donatella.valente@uniroma1.it; 6 Management and Diagnostic Innovations & Clinical Pathways Research Area, Professional Development, Continuous Education and Research Service, Bambino Gesù Children’s Hospital, IRCCS, 00165 Rome, Italy; 7 Department of Life Sciences, Health and Healthcare Professions, Link Campus University, Via del Casale di San Pio V, 44, 00165 Rome, Italy

**Keywords:** amputation, children, hand deficiencies, healthcare service, prosthetics, satisfaction

## Abstract

**Background:** The Child Amputee Prosthetics Project—Prosthesis Satisfaction Inventory (CAPP-PSI) is a comprehensive instrument designed to measure satisfaction across functionality, aesthetic, and service domains. This study aimed to translate, culturally adapt, and evaluate the psychometric properties of the CAPP-PSI in an Italian pediatric population. **Methods**: Following international guidelines, the CAPP-PSI was translated and culturally adapted. Internal consistency was evaluated using Cronbach’s alpha, while test–retest reliability was assessed with intraclass correlation coefficients (ICCs). Construct validity was measured by analyzing correlations among subscales. **Results:** A total of 113 children with congenital or acquired upper limb amputation, accompanied by their parents, were recruited from the Bambino Gesù Children’s Hospital in Rome. The Italian CAPP-PSI demonstrated excellent internal consistency (Cronbach’s alpha = 0.913) and strong test–retest reliability (ICC = 0.966). Subscale correlations showed strong relationships between child and parent satisfaction (r = 0.724, *p* < 0.01) and parent satisfaction with service (r = 0.612, *p* < 0.01), while moderate correlations were observed between child satisfaction and service (r = 0.434, *p* < 0.01). **Conclusions:** The Italian version of the CAPP-PSI is a reliable and valid tool for assessing prosthetic satisfaction in pediatric populations. It provides valuable insights for clinicians and researchers, supporting patient-centered care and targeted improvements in prosthetic design and services. Future studies should explore longitudinal outcomes and the role of psychosocial factors in prosthetic acceptance.

## 1. Introduction

Traumatic amputations in children are relatively uncommon [1], with reported incidence rates ranging from 1.32 to 18.8 per 100,000 individuals [2]. Similarly, congenital limb deficiencies are rare, with unilateral congenital below-elbow deficiencies representing a specific type that affects the forearm and hand. This condition occurs in approximately 1 to 2 live births per 4000 globally [3]. Both traumatic and congenital amputations in children significantly influence daily functionality, independence, and psychosocial well-being. These challenges underscore the critical importance of effective prosthetic devices in promoting rehabilitation and facilitating social integration [4,5]. 

Prosthetic devices can play a crucial role in enhancing motor function, promoting independence, and supporting participation in social and recreational activities. Although the use of upper limb prosthetics can provide notable benefits, research reports high abandonment rates, with an estimated 10% to 49% of pediatric users discontinuing use [6]. Successful prosthetic outcomes rely heavily on both child and parent satisfaction with the device, as satisfaction is directly linked to prolonged use and integration into daily life [7,8]. Without effective functional and aesthetic appeal, children may perceive prosthetic devices as restrictive or burdensome, leading to their rejection. Factors influencing abandonment include discomfort, weight, a lack of sensory feedback, limited control, and maintenance issues [9,10]. Social and psychological factors, such as the desire to fit in with peers or avoid stigmatization, also significantly influence the acceptance and continued use of prostheses [11,12].

The early fitting of prostheses is often recommended to support neural adaptation and motor development. Some studies suggest that prostheses applied within the first year of life may encourage the integration of prosthetic use into natural motor development, while other research indicates that early fitting does not guarantee improved motor outcomes or satisfaction later in life [11,13]. The variability in outcomes underscores the need for comprehensive, individualized assessments of each child’s functional needs, family expectations, and psychosocial context [14].

Given the complexity of factors influencing prosthetic use, validated outcome measures are essential for accurately assessing the impact of prostheses on physical, functional, and psychosocial aspects of life. The International Classification of Functioning, Disability and Health (ICF) framework, recommended by the World Health Organization (WHO), offers a structure for evaluating these multifaceted outcomes [15]. In pediatric prosthetics, specific assessment tools have been designed to evaluate physical and social factors [4], including the Prosthetic Upper Extremity Functional Index (PUFI) [16,17], which assesses the ease of completing tasks with and without the device. The Unilateral Below Elbow Test (UBET) [18], the University of New Brunswick Test of Prosthetic Function (UNB), and the Assessment of Capacity for Myoelectric Control (ACMC) [19] are also frequently used for evaluating motor skills, functionality, and capacity. These tools provide insights into how children interact with prosthetic devices and where challenges may lie. To measure satisfaction, there is a lack of outcome measures in the Italian context; Quebec User Satisfaction with Assistive Technology (QUEST) [20,21] is one of the widely used outcome measure instruments for measuring satisfaction with several assistive products and related services, but this is not specific to measuring prosthetics in pediatrics.

The Child Amputee Prosthetics Project—Prosthesis Satisfaction Inventory (CAPP-PSI) [22] is among the most comprehensive tools for assessing pediatric prosthetic satisfaction. The CAPP-PSI evaluates satisfaction across performance, aesthetics, and service, capturing the perspectives of both children and parents on the device’s usability, appeal, and the quality of prosthetic care provided [22]. The CAPP-PSI includes three main scales: the first assesses satisfaction with the prosthesis’ functionality, the second evaluates satisfaction with appearance, and the third measures satisfaction with the prosthetic team’s service [22]. The inventory has shown high internal consistency and reliability, making it a valuable tool for understanding both the child’s and the family’s experiences with the prosthesis [8]. By providing detailed feedback on satisfaction, the CAPP-PSI supports targeted improvements in prosthetic design and rehabilitation services, ultimately helping to increase device acceptance and enhance quality of life. 

Considering the importance of measuring these issues in clinical practice, the objective of the present investigation is to translate, cross-culturally adapt, and measure the psychometric properties of the CAPP-PSI in an Italian population of children with both acquired and congenital amputations.

## 2. Materials and Methods

### 2.1. Translation and Cross-Cultural Adaptation Process

The initial phase of linguistic and cultural validation followed the methodology of standard translation, back translation, and forward translation, adhering to the guidelines specified in the Principles of Good Practice for the Translation and Cultural Adaptation of Patient-Reported Outcome Measures [23]. Firstly, permission was requested and obtained via email from the authors of the original version, who were also invited to participate in the process. Secondly, two translations from English to Italian (forward translation) were performed by two bilingual professionals, with one being an expert in the field. The two professionals created two separate translations of the instrument, which were then compared and consolidated into a single version through a consensual discussion (reconciliation phase). The produced version was subsequently back-translated into English by two independent native English speakers who were not acquainted with the original scale. These translated versions were then compared with each other and the original version to highlight any discrepancies in wording and the semantic domain (harmonization phase). Next, with the involvement of an occupational therapist, two neuro- and psychomotor therapists, a plastic surgeon, and a physical and rehabilitation medicine doctor, the pre-final version of the CAPP-PSI was applied to a small number of children to verify comprehensibility and interpretation. This aspect was investigated in terms of the semantic domain and wording (cognitive debriefing). Once any discrepancies and semantic equivalence were verified, the research group ultimately obtained the final Italian version of the CAPP-PSI, which was approved and used for the present research.

### 2.2. Participants

According to the established standards for choosing health measurement instruments, a sample size of no fewer than 100 patients is considered suitable for validity assessments [24]. Likewise, for test–retest reliability studies, it is recommended to include at least 50 participants [25]. Participants were recruited from the Department of Intensive Neurorehabilitation and Robotics at the Bambino Gesù Children’s Hospital in Rome between September 2022 and May 2024. Inclusion criteria encompassed children aged 1 to 18 years with congenital or acquired amputation who used a prosthetic device, accompanied by parents or caregivers fluent in Italian. Exclusion criteria comprised children who had undergone hand surgery in the past three months or had visual impairments or other associated medical conditions (orthopedic, neurological, or cognitive disorders).

### 2.3. Measurement Tool

The CAPP-PSI is a structured instrument comprising 14 items, specifically developed to evaluate the level of satisfaction with prosthetic devices among pediatric amputees [22]. This tool encompasses questions designed to gauge perceived satisfaction concerning the fit, functionality, aesthetics, and service of the child’s prosthetic device. It includes three distinct scales for assessment: (1) satisfaction from the perspective of the child, as rated by the parent; (2) parental satisfaction with the prosthetic device’s impact on the child’s daily activities; and (3) parental satisfaction with the service related to the prosthesis [22]. Responses to each item are quantified on a scale ranging from 0 (not at all) to 4 (very much). Each scale on the CAPP-PSI is scored by adding together the scores for each item within the scales. Higher scores indicate greater satisfaction. The items for the CAPP-PSI were generated through a review of the relevant literature in pediatric limb deficiency and through the solicitation of assistance from clinicians with expertise in the area (physicians, physical and occupational therapists, and psychologists) [22]. The final inventory was reviewed for content validity and item clarity by expert clinicians, resulting in 14 face-valid questions to assess prosthesis satisfaction.

### 2.4. Procedures and Data Analysis

In this study, data collection was conducted through a combination of methods. A seasoned occupational therapist engaged with parents through direct interviews and via a digital platform, supplemented by telephone and online methods for the purposes of reliability assessment. Each participant was thoroughly briefed before the commencement of the study and provided written informed consent. Participant details were collated from clinical documentation, interviews with parents, and physical assessments.

For the evaluation of test–retest reliability, participants were instructed to complete the CAPP-PSI a second time within a span of 7 to 15 days following their initial evaluation. This subsequent assessment was carried out through an email-linked web-based platform. Participant sociodemographic and clinical data were quantitatively summarized using descriptive statistics such as frequencies, means, and standard deviations.

This study assessed the reliability of the instrument by examining both its internal consistency and test–retest reliability. Internal consistency was quantified using Cronbach’s alpha, where a threshold of 0.7 was deemed acceptable, as per standard psychometric criteria [24]. Test–retest reliability was evaluated using intraclass correlation coefficients (ICCs), adhering to established interpretative benchmarks [24,25]. 

Furthermore, the construct validity of the CAPP-PSI was analyzed through the correlation of its subscales using the Pearson correlation coefficient. This coefficient (r) measures the strength and direction of linear relationships between continuous variables, with the scale ranging from −1 to 1 [26]. Interpretations of the Pearson coefficient were categorized as follows: 0.40–0.59 indicated a moderate correlation, 0.60–0.79 suggested a strong correlation, and 0.80–1.00 signified a very strong correlation [27]. All statistical analyses were executed using IBM SPSS Statistics software, Version 20.0.

## 3. Results

A total of 113 children (61.1% male and 38.9% female) with a mean (SD) age of 7.80 (6.56) from across the country participated in the study. The majority of the participants had a congenital deficiency (77%) and a metacarpal level of amputation (39.9%) followed by trans-radial amputation (34.5%). Most of the sample used an aesthetic prosthetic device (54.9%), and the mean (SD) age at the first prosthetic prescription was 7.61 (4.27%). Sociodemographic and clinical characteristics are described in Table 1.

The administration of the CAPP-PSI revealed a mean (SD) total score of 28.04 (12.52). The internal consistency study revealed a Cronbach’s coefficient alpha of 0.913 for the total score, 0.858 for the child satisfaction with prosthesis domain, 0.856 for the parent satisfaction with prosthesis domain and 0.920 for the parent satisfaction with service domain. Item–total correlation yielded a Cronbach’s alpha ranging from 0.921 to 0.930 if an item was deleted. Detailed information is reported in Table 2.

The test–retest reliability analysis demonstrated excellent ICC values for the total score, at 0.966 (0.949–0.979), as well as for the three subscales, with ICCs of 0.934, 0.938, and 0.968, respectively, while for construct validity, a moderate-to-strong correlation (0.434–0.612) was found. The results for test–retest reliability and construct validity are synthetized in Table 3 and Table 4, respectively.

## 4. Discussion

The present study aimed to validate the Italian version of the CAPP-PSI in a population of children with congenital or acquired amputation of the upper limb. Our findings revealed good psychometric properties in terms of internal consistency, reliability, and construct validity. 

The Italian translation of the CAPP-PSI followed international recommendations; discrepancies and wording equivalence were investigated in the semantic domain, involving the parents of children with upper limb amputation. From a qualitative point of view, considering that the wording did not include specific medical terms, parents did not report difficulties in understanding, and they felt confident in answering questions. The process we followed guarantees a good understanding of each item and resulted in the final Italian version of the CAPP-PSI (please see Supplementary Materials).

Concerning psychometric properties, our findings showed very good internal consistency (0.966) that exceeds the threshold of 0.70 [28] and is in line with the original validation of the CAPP-PSI [22], which reported an alpha of 0.80 for child satisfaction with prosthesis, an alpha of 0.87 for parent satisfaction with prosthesis, and an alpha of 0.90 for parent satisfaction with service. Cronbach’s alpha serves as a lower-bound estimate of a measure’s reliability and is widely regarded as a conservative indicator [26,29]. Consequently, the alpha coefficients obtained in this study suggest that the items within each scale exhibit internal consistency and collectively measure a single construct. To further evaluate the scales, item–total correlations were computed for each item against the total score of the respective scale. This analysis aimed to assess the strength of the relationship between individual items and the overall scale, ensuring that each item aligns with the construct being measured [30]. Items demonstrating low correlations with the total score would be considered for removal. However, in this study, the item–total correlation was excellent, and, as a result, no items were excluded from the Italian version of the CAPP-PSI based on this criterion. Test–retest reliability also revealed satisfactory values with an ICC of 0.966 for the total score and a range of 0.934–0.968 for CAPP-PSI subscales. This parameter is very important because it refers to the degree to which an outcome measurement instrument consistently produces the same results when administered to the same population under similar conditions at different points in time [31]. It evaluates the stability and reproducibility of measurements across repeated administrations, and our findings revealed that CAPP-PSI is a reliable measure when administered in an interval of 7–15 days.

In terms of construct validity, we found a positive linear correlation with each subscale of the CAPP-PSI; in particular, the child satisfaction–parent satisfaction and parent satisfaction–satisfaction with service subscales exhibited a statistically significant (*p* < 0.01) strong correlation with an r of 0.724 (0.602–0.810) and 0.612 (0.457–0.727), respectively. Child satisfaction–satisfaction with service showed a moderate correlation with an r of 0.434 (0.243–0.590) with a *p* < 0.01. The strong positive correlation between child and parent satisfaction may reflect shared perceptions or experiences regarding the prosthesis, such as its utility, functionality, or aesthetic appeal. It also suggests the interconnected nature of satisfaction within the family context. The strong association between parental satisfaction and satisfaction with service delivery highlights the role of healthcare and service quality in shaping parental perceptions. Parents are likely to attribute their satisfaction not only to the prosthesis itself but also to the quality of interactions with service providers, the adequacy of training or support provided, and the overall care process [32,33]. This finding underscores the importance of service quality as a critical determinant of overall satisfaction in pediatric prosthetic care.

From a qualitative perspective, it can be stated that for both children (as rated by their parents) and parents, the item that received the lowest satisfaction scores was item #1: “Does your child like the way the prosthesis aids in daily activities?” or “Are you happy with the way the prosthesis aids in daily activities?” This finding was unexpected, as evidence exists regarding the positive impact of prosthesis use on performing daily activities [7,34]. However, this result can be explained by the timing of the evaluation, which was conducted just two weeks after prosthesis provision. A recent study found a positive correlation between the number of activities children perform more effectively with their prostheses and the duration of daily use [35,36]. Consequently, satisfaction related to daily activities in our study may be underestimated due to the short assessment timeframe. Furthermore, not all children participated in an intensive rehabilitation program after receiving their prosthesis, particularly those residing in other regions of Italy.

An interesting finding relates to satisfaction with services, with the highest-scoring items being item #9 (“Were you satisfied with the delivery of the prosthesis?”) with a mean score of 2.96, and item #13 (“Are you satisfied with the repair time?”) with a mean score of 2.74. These results are notable given the context of assistive technology provision in Italy, which includes prosthetic services. In March 2017, the Essential Levels of Assistance (LEA) were updated for the first time since 2001, along with the Tariff Nomenclature for Assistive Technology and Prosthetics, which had remained unchanged since 1999 [37,38,39]. Despite these updates, their full implementation has been hindered by the lack of a necessary tariff decree, effectively freezing the system in a framework over 20 years old. Furthermore, recent legislation has shifted toward a tender-based system aimed at cost efficiency. While it potentially reduces expenses, this approach risks compromising the customization of devices, thereby affecting their alignment with individual user needs. Additionally, inconsistencies in how the updated Nomenclature is interpreted and applied across Regional Health Services have created disparities in the provision of aids and prosthetics between regions. These regional variations risk creating inequities in access to essential devices. The high satisfaction reported in our hospital may reflect an unexpectedly efficient and personalized service delivery model, which contrasts with broader systemic challenges, thus explaining the positive feedback from users.

Despite these encouraging results, there is a need to acknowledge some limitations. First, there is the aforementioned timing of the satisfaction evaluation: an evaluation two weeks post-prosthesis provision may have underestimated the level of satisfaction with the prosthesis in daily activities. Previous research suggests that satisfaction increases with prolonged use and as users adapt to the prosthesis [5,22]. A follow-up evaluation after a longer period, ideally several months post-provision, would provide a more accurate measure of satisfaction and functional integration. Second, while this study assessed satisfaction and functional outcomes, it did not comprehensively evaluate psychosocial factors, such as peer interactions, self-esteem, or coping strategies. These aspects are crucial in understanding the broader impact of prosthetic use, particularly in pediatric populations. Future studies should incorporate validated tools to capture these dimensions. Third, the study did not extensively control for potential confounders, such as the type and quality of rehabilitation programs, socioeconomic factors, or parental education levels. These factors could significantly influence satisfaction outcomes and should be accounted for in future analyses. Lastly, the cross-sectional nature of this study limits the ability to assess how satisfaction, functionality, and psychosocial outcomes evolve over time. Longitudinal studies are needed to track these outcomes and identify factors that influence prosthetic acceptance or abandonment in the long term.

## 5. Conclusions 

In conclusion, the CAPP-PSI was found to be a reliable and valid tool for measuring satisfaction with prosthetic provision in children with upper limb amputation. This assessment tool can evaluate both functional outcomes and satisfaction, guiding clinicians in refining device selection, fitting, and support. Through the use of the CAPP-PSI, practitioners can adopt a patient-centered approach, fostering adherence and improving the well-being of children with upper limb deficiencies. Moreover, the findings underscore the importance of comprehensive assessment tools in pediatric prosthetic care, ensuring that interventions are both effective and attuned to the patients’ and families’ needs. Future research should aim to expand upon these findings by exploring long-term outcomes and the impact of psychosocial factors on prosthetic use, which could further refine clinical practices and improve quality of life for these children. With the CAPP-PSI, clinicians have a robust instrument to measure and improve patient satisfaction and outcomes in prosthetic care, contributing to better healthcare delivery and patient experiences in pediatric rehabilitation.

## Figures and Tables

**Table 1:** Sociodemographic and clinical characteristics of the sample (total: 113).

**Age Mean (SD)**	7.80 (6.56)	
**Gender**	Frequency	%
Male	69	61.1
Female	44	38.9
**Amputation**	Frequency	%
Acquired	26	23.0
Congenital	87	77.0
**Level of Amputation**	Frequency	%
Metacarpus	45	39.9
Carpus	18	15.9
Trans-radial	30	34.5
Trans-humeral	11	9.7
**Prosthetic Prescription Mean (SD)**	7.61 (4.27)	
**Type of Prostheses**	Frequency	%
Aesthetic	62	54.9
Functional (body- or myoelectric-powered)	51	45.1
**Regional Distribution**	Frequency	%
Northern Italy	44	38.94
Central Italy	43	38.05
Southern Italy and Islands	26	23.01

**Table 2:** Item–total statistics for the 14 items of the CAPP-PSI.

		Mean (SD) Score	Scale Mean If Item Deleted	Scale Variance If Item Deleted	Corrected Item–Total Correlation	Squared Multiple Correlation	Cronbach’s Alpha If Item Deleted
**Parent-rated child satisfaction with prosthesis**							
1	Does your child like the way the prosthesis aids in daily activities	1336 (1.16)	26,708	138,066	0.642	0.811	0.927
2	Does your child like the way the prosthesis fits	1796 (1.17)	26,248	137,402	0.660	0.824	0.926
3	Does your child like the way the prosthesis functions	1407 (1.15)	26,637	137,305	0.677	0.835	0.926
4	Does your child like the appearance of the prosthesis	1867 (1.28)	26,177	139,129	0.533	0.694	0.930
**Parent satisfaction with prosthesis**							
5	Are you happy with the way the prosthesis aids in daily activities	1336 (1.16)	26,708	136,244	0.714	0.789	0.925
6	Are you happy with the way the prosthesis fits	1832 (1.16)	26,212	134,044	0.800	0.837	0.922
7	Are you happy with the way the prosthesis functions	1522 (1.15)	26,522	135,377	0.751	0.853	0.923
8	Are you happy with the appearance of the prosthesis	2009 (1.39)	26,035	136,427	0.569	0.759	0.930
**Parent satisfaction with service**							
9	Were you satisfied on delivery of the prosthesis	2956 (0.93)	25,088	142,599	0.601	0.709	0.928
10	Are you satisfied with follow-up care	2204 (1.38)	25,841	130,439	0.779	0.848	0.922
11	Are you satisfied with instruction provided	2363 (1.46)	25,681	128,273	0.800	0.846	0.921
12	Are you satisfied with the manufacture time	2558 (1.24)	25,487	135,252	0.695	0.765	0.925
13	Are you satisfied with the repair time	2743 (1.12)	25,301	140,802	0.558	0.620	0.929
14	Are you satisfied with the child’s training	2115 (1.36)	25,929	133,406	0.690	0.704	0.925

**Table 3:** Test–retest reliability of the CAPP-PSI.

CAPP-PSI Subscales	ICC	Lower Bound	Upper Bound
Child satisfaction with prosthesis (parent-rated)	0.934	0.899	0.960
Parent satisfaction with prosthesis	0.938	0.906	0.963
Parent satisfaction with service	0.968	0.952	0.981
Total score	0.966	0.949	0.979

**Table 4:** Construct validity of the CAPP-PSI.

CAPP-PSI Subscales	r	Sig	Lower Bound	Upper Bound
Child satisfaction–Parent satisfaction	0.724	<0.01	0.602	0.810
Child satisfaction–Satisfaction with service	0.434	<0.01	0.243	0.590
Parent satisfaction–Satisfaction with service	0.612	<0.01	0.457	0.727

## Data Availability

Data are available from the corresponding author upon reasonable request due to privacy.

## Supplementary Materials

The following supporting information can be downloaded at: https://www.mdpi.com/article/10.3390/children12020130/s1. Italian version of the CAPP-PSI.

## References

[1] Abzug J.M., Kozin S.H. (2014). Pediatric Replantation. J. Hand Surg. Am..

[2] Vakhshori V., Bouz G.J., Mayfield C.K., Alluri R.K., Stevanovic M., Ghiassi A. (2019). Trends in Pediatric Traumatic Upper Extremity Amputations. HAND.

[3] Tonkin M.A. (2017). Classification of Congenital Anomalies of the Hand and Upper Limb. J. Hand Surg. (Eur. Vol.).

[4] Lindner H.Y.N., Nätterlund B.S., Hermansson L.M.N. (2010). Upper Limb Prosthetic Outcome Measures. Prosthet. Orthot. Int..

[5] Manocchio N., Gaudenzi M., Tofani M., Ljoka C., Imeshtari A., Giordani L., Della Bella G., Foti C. (2024). Functional Impact of Early Prosthetic Implantation in Children with Upper Limb Agenesis or Amputation. Appl. Sci..

[6] Biddiss E.A., Chau T.T. (2007). Upper Limb Prosthesis Use and Abandonment. Prosthet. Orthot. Int..

[7] Sims T., Donovan-Hall M., Metcalf C. (2020). Children’s and Adolescents’ Views on Upper Limb Prostheses in Relation to Their Daily Occupations. Br. J. Occup. Ther..

[8] Cordella F., Ciancio A.L., Sacchetti R., Davalli A., Cutti A.G., Guglielmelli E., Zollo L. (2016). Literature Review on Needs of Upper Limb Prosthesis Users. Front. Neurosci..

[9] Vasluian E., de Jong I.G.M., Janssen W.G.M., Poelma M.J., van Wijk I., Reinders-Messelink H.A., van der Sluis C.K. (2013). Opinions of Youngsters with Congenital Below-Elbow Deficiency, and Those of Their Parents and Professionals Concerning Prosthetic Use and Rehabilitation Treatment. PLoS ONE.

[10] Michielsen A., Van Wijk I., Ketelaar M. (2010). Participation and Quality of Life in Children and Adolescents with Congenital Limb Deficiencies. Prosthet. Orthot. Int..

[11] Meurs M., Maathuis C.G.B., Lucas C., Hadders-Algra M., van der Sluis C.K. (2006). Prescription of the First Prosthesis and Later Use in Children with Congenital Unilateral Upper Limb Deficiency. Prosthet. Orthot. Int..

[12] Marinković B., Ćorluka B., Vukajlović M., Bjelica B., Aksović N., Bubanj S., Petković E., Preljević A., Lilić L., Dobrescu T. (2024). The Relationship Between Psychological Factors and Nutritional Status in Adolescence. Children.

[13] Huizing K., Reinders-Messelink H., Maathuis C., Hadders-Algra M., van der Sluis C.K. (2010). Age at First Prosthetic Fitting and Later Functional Outcome in Children and Young Adults with Unilateral Congenital Below-Elbow Deficiency. Prosthet. Orthot. Int..

[14] Buffart L.M., Roebroeck M.E., Pesch-Batenburg J.M.F.B., Janssen W.G.M., Stam H.J. (2006). Assessment of Arm/Hand Functioning in Children with a Congenital Transverse or Longitudinal Reduction Deficiency of the Upper Limb. Disabil. Rehabil..

[15] Tofani M., Mustari M., Tiozzo E., Dall’Oglio I., Morelli D., Gawronski O., Salata M., Cantonetti L., Castelli E., Di Lallo D. (2023). The Development of the International Classification of Functioning, Disability and Health for Child and Youth (ICF-CY) Core Sets: A Systematic Review. Disabil. Rehabil..

[16] Virginia Wright F., Hubbard S., Jutai J., Naumann S. (2001). The Prosthetic Upper Extremity Functional Index: Development and Reliability Testing of a New Functional Status Questionnaire for Children Who Use Upper Extremity Prostheses. J. Hand Ther..

[17] Wright F.V., Hubbard S., Naumann S., Jutai J. (2003). Evaluation of the Validity of the Prosthetic Upper Extremity Functional Index for Children. Arch. Phys. Med. Rehabil..

[18] Bagley A.M., Molitor F., Wagner L.V., Tomhave W., James M.A. (2006). The Unilateral Below Elbow Test: A Function Test for Children with Unilateral Congenital below Elbow Deficiency. Dev. Med. Child Neurol..

[19] Burger H., Brezovar D., Vidmar G. (2014). A Comparison of the University of New Brunswick Test of Prosthetic Function and the Assessment of Capacity for Myoelectric Control. Eur. J. Phys. Rehabil. Med..

[20] Galeoto G., Colucci M., Guarino D., Esposito G., Cosma E., De Santis R., Grifoni G., Valente D., Tofani M. (2018). Exploring Validity, Reliability, and Factor Analysis of the Quebec User Evaluation of Satisfaction with Assistive Technology in an Italian Population: A Cross-Sectional Study. Occup. Ther. Health Care.

[21] Berardi A., Galeoto G., Lucibello L., Panuccio F., Valente D., Tofani M. (2020). Athletes with Disability’ Satisfaction with Sport Wheelchairs: An Italian Cross Sectional Study. Disabil. Rehabil. Assist. Technol..

[22] Pruitt S.D., Varni J.W., Seid M., Setoguchi Y. (1997). Prosthesis Satisfaction Outcome Measurement in Pediatric Limb Deficiency. Arch. Phys. Med. Rehabil..

[23] Wild D., Grove A., Martin M., Eremenco S., McElroy S., Verjee-Lorenz A., Erikson P. (2005). Principles of Good Practice for the Translation and Cultural Adaptation Process for Patient-Reported Outcomes (PRO) Measures: Report of the ISPOR Task Force for Translation and Cultural Adaptation. Value Health.

[24] Terwee C.B., Bot S.D.M., de Boer M.R., van der Windt D.A.W.M., Knol D.L., Dekker J., Bouter L.M., de Vet H.C.W. (2007). Quality Criteria Were Proposed for Measurement Properties of Health Status Questionnaires. J. Clin. Epidemiol..

[25] Lexell J.E., Downham D.Y. (2005). How to Assess the Reliability of Measurements in Rehabilitation. Am. J. Phys. Med. Rehabil..

[26] Hundleby J.D., Nunnally J. (2006). Psychometric Theory. Am. Educ. Res. J..

[27] Monticone M., Galeoto G., Berardi A., Tofani M. (2021). Psychometric Properties of Assessment Tools. Measuring Spinal Cord Injury.

[28] Ratner B. (2009). The Correlation Coefficient: Its Values Range between 1/1, or Do They. J. Target. Meas. Anal. Mark..

[29] Tavakol M., Dennick R. (2011). Making sense of Cronbach’s alpha. Int. J. Med. Educ..

[30] Tang W., Cui Y., Babenko O. (2014). Internal Consistency: Do We Really Know What It Is and How to Assess It?. J. Psychol. Behav. Sci..

[31] Zijlmans E.A.O., Tijmstra J., van der Ark L.A., Sijtsma K. (2019). Item-Score Reliability as a Selection Tool in Test Construction. Front. Psychol..

[32] Lakshmi S., Akbar Mohideen M. (2013). Issues in reliabilityand validity of research. Int. J. Manag. Res. Rev..

[33] Utami F., Argianto A. (2023). Perceived Service Quality and User Satisfaction with Orthotic-Prosthetic Devices and Services Among Individual with Physical Disabilities. IJDS Indones. J. Disabil. Stud..

[34] Oliver J., Dixon C., Murray C.D. (2020). Being the Parent of a Child with Limb Difference Who Has Been Provided with an Artificial Limb: An Interpretative Phenomenological Analysis. Disabil. Rehabil..

[35] van Dijk-Koot C.A., van der Ham I., Buffart L.M., van der Sluis C.K., Stam H.J., Pesch-Batenburg J.M.F.B., Roebroeck M.E. (2009). Current Experiences With the Prosthetic Upper Extremity Functional Index in Follow-Up of Children With Upper Limb Reduction Deficiency. JPO J. Prosthet. Orthot..

[36] Mano H., Noguchi S., Fujiwara S., Haga N. (2023). Relationship between Degree of Disability, Usefulness of Assistive Devices, and Daily Use Duration: An Investigation in Children with Congenital Upper Limb Deficiencies Who Use Upper Limb Prostheses. Assist. Technol..

[37] Presidenza del Consiglio dei Ministri Decreto del Presidente del Consiglio dei Ministri (DPCM) del 12 Gennaio 2017. Gazzetta Ufficiale della Repubblica Italiana, Serie Generale n.65 del 18 marzo 2017—Supplemento Ordinario n. 15. https://www.gazzettaufficiale.it/eli/id/2017/03/18/17A02015/sg.

[38] Ministero della Salute I Livelli Essenziali di Assistenza (LEA): Informazioni Generali e Dettagli. https://www.salute.gov.it/portale/lea/dettaglioContenutiLea.jsp?area=Lea&id=4773&menu=vuoto.

[39] Ministero della Salute Nomenclatore Dell’assistenza Protesica: Aggiornamento DPCM 12 Gennaio 2017. https://www.salute.gov.it/portale/programmazioneFinanziamentoSSN/dettaglioContenutiProgrammazioneFinanziamentoSSN.jsp?area=programmazioneSanitariaLea&id=1312&menu=tariffari.

