# Supplementary material for: Child Amputee Prosthetics Project—Prosthesis Satisfaction Inventory (CAPP-PSI): Validation of Italian Version in Children with Upper Limb Amputation
Source: Children (Basel). 2025 Jan 24;12(2):130. doi: 10.3390/children12020130 (PMC11854260; doi:10.3390/children12020130)
Supplement: Supplementary file 1 [file children-12-00130-s001.zip › children-3397629-supplementary.pdf]

CAPP-PSI-IT

Percorso di Validazione per la versione italiana

Child Amputee Prosthetics Project – Prosthesis Satisfaction Inventory (CAPP PSI)

Si prega di valutare ognuna delle voci seguenti da una scala da 0 a 4

0= 'per niente'	1= 'leggermente'	2= 'abbastanza'	3= 'molto'	4 = 'estremamente'

**Soddisfazione del bambino (Giudizio genitore)**	**0 (per niente) _________ 4 (estremamente)**				
Al bambino piace il modo in cui la protesi lo aiuta nelle attività di vita quotidiana?	0	1	2	3	4
Al bambino piace il modo in cui veste/si adatta la protesi?	0	1	2	3	4
Al bambino piace il modo in cui la protesi funziona?	0	1	2	3	4
Al bambino piace l'aspetto /estetica della protesi?	0	1	2	3	4

Soddisfazione del genitore rispetto alla protesi

Lei è soddisfatto del modo in cui la protesi lo aiuta nelle attività di vita quotidiana?	0	1	2	3	4
Lei è soddisfatto del modo in cui veste/si adatta la protesi?	0	1	2	3	4
Lei è soddisfatto del modo in cui la protesi funziona?	0	1	2	3	4
Lei è soddisfatto dell'aspetto/estetica della protesi?	0	1	2	3	4

Soddisfazione del genitore rispetto al servizio

Lei è soddisfatto del servizio di consegna della protesi?	0	1	2	3	4
Lei è soddisfatto dei successivi controlli?	0	1	2	3	4
Lei è soddisfatto delle istruzioni/indicazioni fornite?	0	1	2	3	4
Lei soddisfatto dei tempi di fabbricazione?	0	1	2	3	4
Lei è soddisfatto dei tempi di riparazione?	0	1	2	3	4
Lei è soddisfatto delle attività di riabilitazione/training rispetto all'utilizzo della protesi?	0	1	2	3	4

____

Total
